# Endodontic Treatment in Submerged Roots: A Case Report

**DOI:** 10.5681/joddd.2010.017

**Published:** 2010-06-24

**Authors:** Hemalatha Pameshwar Hiremath, Yogesh S. Doshi, Sadanand Siddayya Kulkarni, Saurav Kumar Purbay

**Affiliations:** ^1^ Senior Lecturer, Department of Endodontics, Rural Dental College, Loni, India; ^2^ Senior Lecturer, Department of Periodontics, Deen Dayal Dental College, Sholapur, India; ^3^ Professor, Head of the Department of Pedodontics, Rural Dental College, Loni, Ahmadnagar India; ^4^ Senior Lecturer, Department of Endodontics, Rural Dental College, Loni, India

**Keywords:** Alveolar bone resorption, endodontic treatment, retained roots, root submergence

## Abstract

Alveolar ridge resorption has long been considered an unavoidable consequence of tooth extraction. While the extent and pattern of resorption is variable among individuals, there is a progressive loss of ridge contour as a result of physiologic bone remodeling. Even today, with best modalities of tooth preservation, there is a group of elderly individuals who do not beneﬁt from modern preventive practices and who now present a dilemma in terms of maintaining the masticatory apparatus necessary for nutrition. Even with excellent dental care, such patients experience abrasion of the natural tooth crowns with age, and embedded roots are left within the alveolar bone. According to old concepts of dental care, extraction of these roots would have been recommended, but today’s goal of excellence in endodontics dictates otherwise. We report a case in which vital and non-vital root submergence was carried out to prevent alveolar ridge reduction.

## Introduction

Edentulism was once considered normal for anyone in their seventh decade of life, as was so eloquently declaimed by Jaques in Shakespeare’s ‘As You Like It’: “sans teeth, sans eyes, sans taste, sans everything.”

Alveolar bone resorption has been defined as “a complex multifactorial oral disease governed by physical and physiologic laws”. Little progress has been made concerning the etiology, treatment, and prevention of this disease. However, the one fact that cannot be disputed is that alveolar bone reduction is progressive and irreversible following tooth extraction.^1^ Atwood and Coy found the mean reduction for the anterior maxillary to be a loss of 1 mm per year and for the anterior mandible, 0.4 mm per year. The only reliable method known to preserve alveolar bone is the maintenance of functioning healthy teeth.^2^

In the practice of removable prosthodontics, much attention is given to the preservation of the residual ridge. Regardless of what material, technique, or philosophy is used, the fate of the supportive bone is a major factor in the success of any denture. Bone continues to be a dynamic tissue that responds to function. The extraction of teeth eliminates the need for an alveolar process, and the bone is resorbed.^3^

The overdenture concept, which was developed in an effort to preserve alveolar bone by retaining natural teeth, has become an accepted technique. However, the disadvantages of caries, periodontal disease, and high cost have plagued the overdenture concept. It was because of these disadvantages that a simpler method of root retention was sought, resulting in the development of the submerged root concept.^4^

The genesis of the submerged root concept probably evolved from roots fractured and left behind during extraction, which may be retained in the alveolar bone with no evidence of pathosis.^1^

We report a case in which vital and non-vital root submergence was carried out to prevent the alveolar ridge reduction.

## Case Report

A 70-year-old man reported to the Department of Prosthodontics for denture placement. He was referred to the Department of Conservative Dentistry and Endodontics for consultation regarding the teeth which required endodontic re-treatment procedures, and to assess the periapical status of remaining teeth. Radiographs revealed several teeth which required root canal re-treatment procedures and one tooth requiring fresh root canal treatment (Figure 1).

**Figure:**
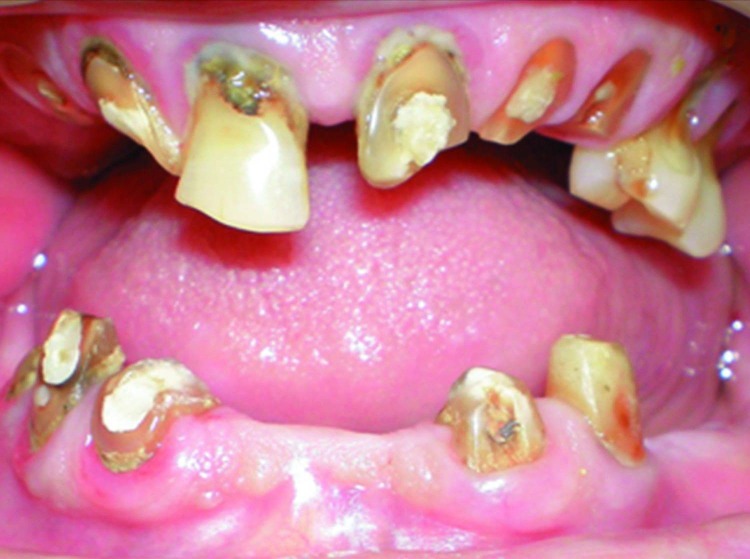
Figure 1. Pre-operative clinical photograph.

Clinical examination revealed periodontal involvement of teeth in both arches and several missing teeth (Figure 2). During presentation of the treatment plan several teeth were advised to be extracted. Later the patient was briefed about root submergence which would prevent the problem of bone resorption associated with denture placement. When the patient showed interest in the proposed treatment plan, non-vital and vital root submergence was elected. Extraction of periodontally involved teeth was carried out, namely #16, #17, #24, #25, #26, #27, and #37. Subsequently, endodontic procedure was performed on #11; re-treatment endodontic procedure was performed with the remaining seven teeth: #12, #13, #22, #23, #32, #42, and #43. After endodontic therapy the patient was followed for a month to verify the root canal procedures. Vital and non-vital root submergence procedure was carried on, by raising a full thickness flap (Figure 3). The coronal structure of the teeth selected for vital (#33, #21) and non-vital submergence (#11, #12, #13, #22, #23, #32, #42, and #43) was reduced to 2 mm below the alveolar crest. After reduction, gutta-percha was burnished with a ball burnisher. The flap was sutured using resorbable suture and primary closure obtained. Healing was uneventful and a 3-month recall visit revealed complete tissue coverage and good prognosis of endodontic therapy. The patient was subsequently scheduled to have his denture placed. The patient was evaluated after 18 months. Radiographically, most of the retained roots appeared normal with regard to surgical success, periodontal support, lamina dura presence and absence of periapical pathosis (Figure 4).

**Figure:**
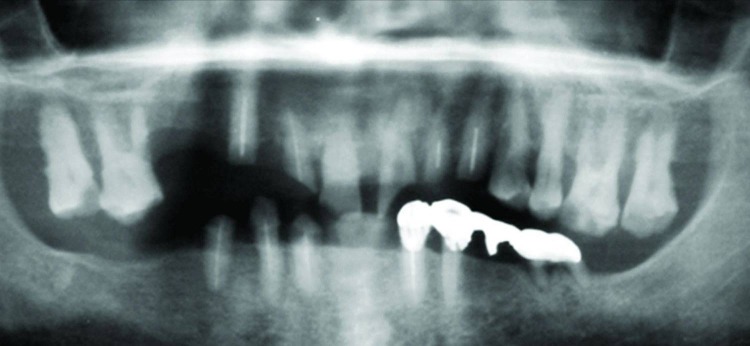
Figure 2. Pre-operative panoramic view.

**Figure:**
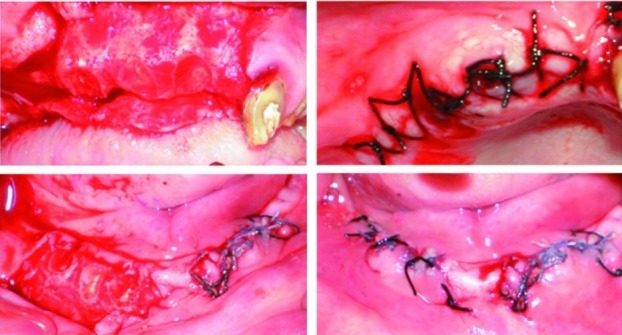
Figure 3. Maxillary and mandibular arches during vital and non-vital root submergence procedures.

**Figure:**
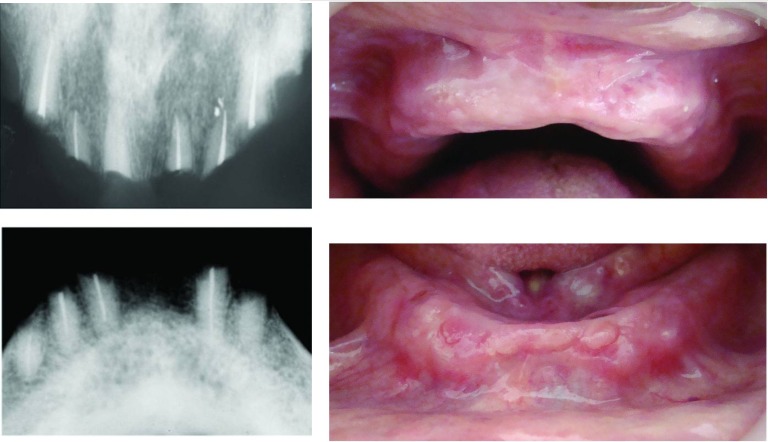
Figure 4. Follow-up radiograph after 18 months showing healthy periapical area. Clinical pictures after 18 months of vital and non-vital root submergence.

## Discussion

Atwood^5^ observed that the “Reduction of residual ridges needs to be recognized for what it is: A major unsolved oral disease which causes physical, psychologic, and economic problems for millions of people all over the world.” Both objective and subjective findings clearly indicate the significant benefits of tooth retention since even the extraction of a patient’s few remaining teeth should be a serious decision.^6^ The success of complete denture service is predictably based on the maintenance of the integrity of supportive oral tissues. Alveolar bone maintenance depends on the presence of healthy roots and periodontal ligaments, which transmit functional and parafunctional forces to the surrounding bone. The loss of teeth and periodontal ligaments and their replacement by complete dentures inevitably changes the pattern of force distribution.^7^

Denture pressure on a residual ridge also results in bone resorption. However, when tensile stresses are received by bone, additional bone is formed. Such stresses occur when occlusal forces are transmitted to the alveolus by the periodontal ligament. This latter principle has been used by prosthodontists in an attempt to preserve the alveolar bone, not only about teeth with advanced periodontal disease for which fixed splints are used but also about retained pulpless roots that contribute to the support of a complete denture.^3^

In 1959, Simpson^8^ examined a number of symptom-free retained roots containing healthy pulp tissue in humans. His evidence suggests that root fragments which were originally uninfected could be safely left in position. It was reported by Poe^9^ and associates that the mucosal coverage of vital roots in three dogs resulted in: 1. Maintenance of vitality of all roots up to 4 months; 2. Calcifications in some canals; and 3. No detectable pathologic changes.

In 1960 Helsham^10^ in a survey of 2000 patients with retained roots found that 1676 patients were without symptoms or pathoses. He also noted that the histologic examination of 60 root fragments showed 46 ones to contain vital pulp tissue; one was non-vital and the remainder was sclerosed bone or cementum. In a 1973 study of 228 retained root tips, Herd^11^ found that 163 had vital pulp tissue with no inflammation.

Bjorn^12^ was the first person to publish a report of root submersions. Jhonson^13^ in 1979 submerged 36 vital teeth in 10 patients and followed the vitality and position of the sectioned roots, the surface integrity of soft tissue coverage, and the osseous tissue character surrounding the roots of the sectioned teeth for 3 years. He concluded that the patients in general felt as though they had some of their own teeth, which suggests more of an intact body image, and exhibited good proprioceptive, perceptive and psychologic response. Similar technique of submergence involving canine roots beneath complete denture was reported by Murray and Adkins^14^ during the same year. Their observation indicated that this technique provides a practical means of retaining the alveolar bone. Ortega Alejandra and Salgado Silva^15^ in 1991 concluded that atrophy of the alveolar process can be avoided by intentionally preserving dental roots in patients with ideal periodontal and pulpal health conditions.

The concept of vital root retention was also proposed by Von Wowern and Winther^16^ in 1981, based on the observation that bone resorption did not occur around retained teeth, but this was later abandoned due to soft tissue complications.

Rodd et al^17^ in 2002 justified the efforts to retain permanent anterior roots in a young population in light of the high clinical success rate of over 90% over a 2-year period.

In 2007 Maurice Salama et al^18^ suggested a strategy to provide a more predictable protocol for esthetic implant treatment for multiple-tooth defects using the root submergence technique (RST). RST maintains the natural attachment apparatus of the tooth in the pontic area, which in turn allows for complete preservation of the alveolar bone frame and assists in the creation of an esthetic result in adjacent multiple tooth replacement cases.

Force exerted on the alveolar bone from the complete denture base is in the form of pressure, which is unfavorably tolerated by the alveolar bone. The technique of tooth root retention under complete dentures appears to militate against such a force application. The evaluation of individual teeth for the presence or absence of pulpal involvement is essential to the success of root submergence.

In the case report presented several teeth were selected for re-treatment of endodontic procedures, and a single tooth was selected for fresh root canal therapy, which later accounted for non-vital submergence, and two teeth were selected for vital submergence. In these two teeth the coronal part of the submerged roots were not sealed with any material as the patient was advised pre-procedural rinse with chlorhexidine digluconate 0.2% for 1 minute^19^ and water-tight sutures were placed immediately to achieve isolation of the vital pulp from the oral bacteria. Studies have shown that use of chlorhexidine reduces the bacterial load up to 95% and in the absence of microleakage the pulp will have the highest probability for wound repair and survival.^20,21^ Proper diagnosis and adequate endodontic treatment of submucosally retained roots lead to an excellent tissue acceptance and ridge preservation. Three-month follow-up revealed excellent healing of soft tissues. The oral tissues appeared normal in color and texture, and the denture remained stable and retentive. The patient reported favorable denture experience. Clinical examination revealed no further reduction of the residual ridge in the region of covered roots.

## Conclusion

It can be concluded that mucosal coverage of roots as a means of preserving the residual alveolar ridge is a sound clinical method for those patients where overdenture is not possible and could be a viable option to complete extractions. The undisturbed root attached to the alveolar bone by the periodontal ligament is the “perfect” implant.
